# Organic Cation Transporters in Human Physiology, Pharmacology, and Toxicology

**DOI:** 10.3390/ijms21217890

**Published:** 2020-10-24

**Authors:** Sophia L. Samodelov, Gerd A. Kullak-Ublick, Zhibo Gai, Michele Visentin

**Affiliations:** 1 Department of Clinical Pharmacology and Toxicology, University Hospital Zurich, University of Zurich, 8006 Zurich, Switzerland; sophia.samodelov@usz.ch (S.L.S.); gerd.kullak@usz.ch (G.A.K.-U.); 2 Mechanistic Safety, CMO & Patient Safety, Global Drug Development, Novartis Pharma, 4056 Basel, Switzerland; 3 Experimental Center, Shandong University of Traditional Chinese Medicine, Jinan 250355, China

**Keywords:** hepatotoxicity, nephrotoxicity, organic cation transporter, solute carrier

## Abstract

Individual cells and epithelia control the chemical exchange with the surrounding environment by the fine-tuned expression, localization, and function of an array of transmembrane proteins that dictate the selective permeability of the lipid bilayer to small molecules, as actual gatekeepers to the interface with the extracellular space. Among the variety of channels, transporters, and pumps that localize to cell membrane, organic cation transporters (OCTs) are considered to be extremely relevant in the transport across the plasma membrane of the majority of the endogenous substances and drugs that are positively charged near or at physiological pH. In humans, the following six organic cation transporters have been characterized in regards to their respective substrates, all belonging to the solute carrier 22 (SLC22) family: the organic cation transporters 1, 2, and 3 (OCT1–3); the organic cation/carnitine transporter novel 1 and 2 (OCTN1 and N2); and the organic cation transporter 6 (OCT6). OCTs are highly expressed on the plasma membrane of polarized epithelia, thus, playing a key role in intestinal absorption and renal reabsorption of nutrients (e.g., choline and carnitine), in the elimination of waste products (e.g., trimethylamine and trimethylamine N-oxide), and in the kinetic profile and therapeutic index of several drugs (e.g., metformin and platinum derivatives). As part of the Special Issue Physiology, Biochemistry, and Pharmacology of Transporters for Organic Cations, this article critically presents the physio-pathological, pharmacological, and toxicological roles of OCTs in the tissues in which they are primarily expressed.

## 1. Introduction

**Figure 1:**
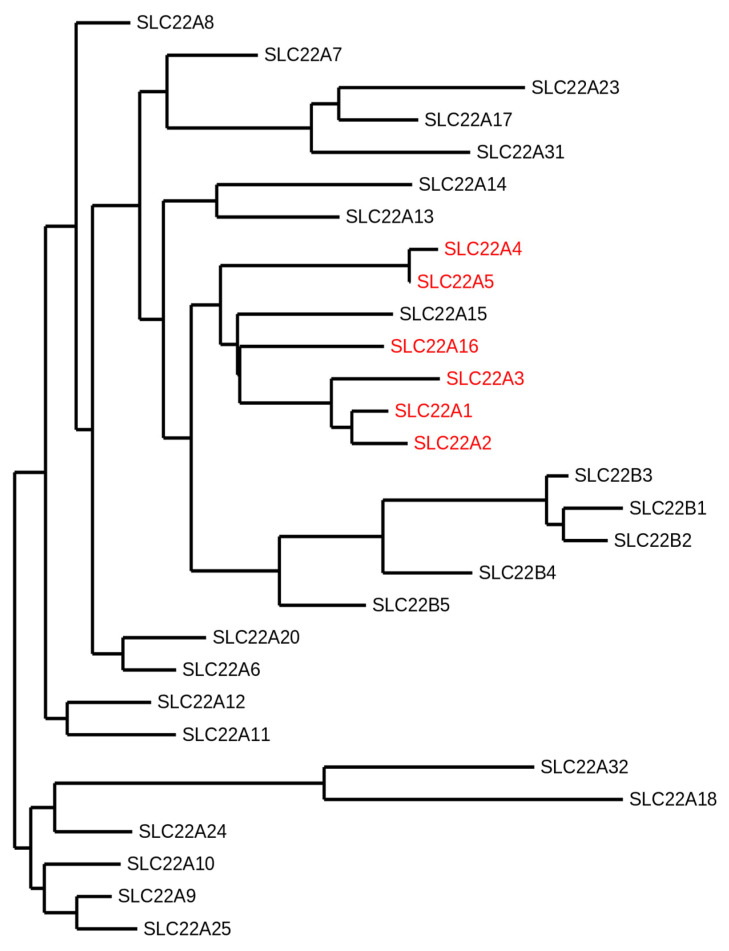
Phylogram of the human solute carrier 22 (SLC22) family members. The following protein sequence were used: SLC22A1 (O15245.2), SLC22A2 (O15244.2), SLC22A3 (O75751.1), SLC22A4 (Q9H015.3), SLC22A5 (O76082.1), SLC22A6 (Q4U2R8.1), SLC22A7 (Q9Y694.1), SLC22A8 (Q8TCC7.1), SLC22A9 (Q8IVM8.1), SLC22A10 (Q63ZE4.2), SLC22A11 (Q9NSA0.1), SLC22A12 (Q96S37.1), SLC22A13 (Q9Y226.2), SLC22A14 (Q9Y267.4), SLC22A15 (Q8IZD6.1), SLC22A16 (Q86VW1.1), SLC22A17 (Q8WUG5.1), SLC22A18 (Q96BI1.3), SLC22A20 (A6NK97.1), SLC22A23 (A1A5C7.2), SLC22A24 (Q8N4F4.2), SLC22A25 (Q6T423.2), SLC22A31 (A6NKX4.4), SLC22A32 (Q14728.1), SLC22B1 (Q7L0J3.1), SLC22B2 (Q7L1I2.1), SLC22B3 (Q496J9.1), SLC22B4 (Q8N4V2.1), and SLC22B5 (Q8N434.2). The organic cation transporters are highlighted in red. The SLC22A15 transporter that clusters with the other organic cation transporters has not been functionally characterized yet. This phylogeny was generated using the open access software Phylogeny.fr [9,10,11].

The organic cation transporters are primarily members of the solute carrier 22 (SLC22) family, which itself belongs to the solute carrier (SLC) superfamily, the largest group of membrane transporters comprising 65 SLC families (SLC1–65) with more than 400 identified genes thus far (for details on the SLC classification, we refer to the curated BioParadigms.org online SLC table) [1]. SLCs regulate the transport of most of the molecules essential for cell life across biomembranes and they have been linked to more than a hundred monogenic disorders [2]. In the human SLC22 family, six organic cation transporters have been characterized in regard to their respective substrates. The organic cation transporters 1, 2, and 3 (OCT1–3) are encoded by the genes *SLC22A1*, -*2*, and -*3*. The organic cation/carnitine transporter novel 1 and 2 (OCTN1 and N2) and the organic cation transporter 6 (OCT6) are encoded by the *SLC22A4*, -*5*, and -*16*, respectively [3]. Other members of the human SLC22 family comprise eight anion transporters (OATs), one urate transporter (URAT1), and fourteen orphan proteins, as no substrate thereof has yet been identified (Figure 1) [1]. Phylogenetic analyses suggest that SLC22 transporters may have evolved over 450 million years ago, with putative SLC22 orthologues found in worms, sea urchins, flies, and ciona [4]. The transporters discussed in this review are the highly related members OCT1–3 and OCTN1–2, as well as the more recently characterized OCT6, encoded by *SLC22A16* and cloned alongside with *SLC22A15* in 2002. Because no substrate of the latter has yet been identified, it will not be discussed in this review (see [5] Eraly et al., [6] Okada et al., [7] Zhu et al., and [8] Drake et al. for the current status of information on human SLC22A15).

**Figure 2:**
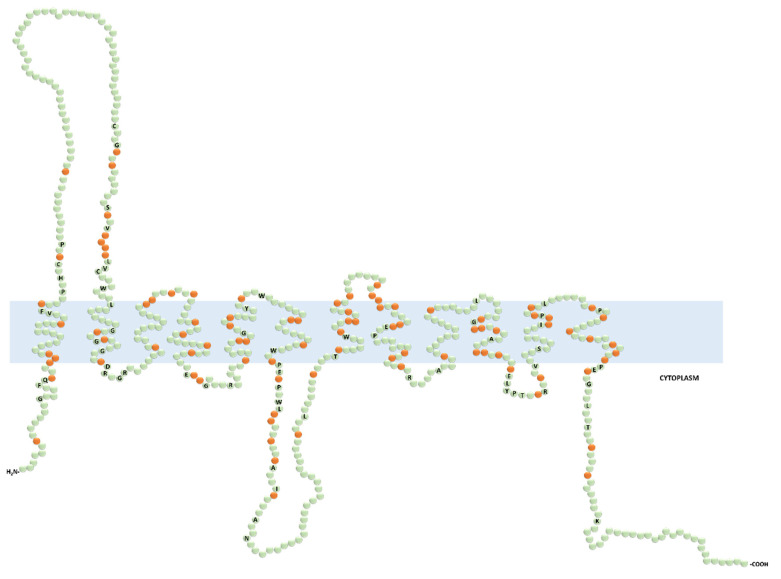
Predicted secondary structure of the functionally characterized human organic cation transporters (OCTs). Prediction was generated with the Protter open access software from the input protein sequence Q86VW1.1 (OCT6) and aligned by CLUSTALW open access software with the following protein sequences: SLC22A1 (O15245.2), SLC22A2 (O15244.2), SLC22A3 (O75751.1), SLC22A4 (Q9H015.3), and SLC22A5 (O76082.1). The labeled and non-labeled residues in green color represent the fully conserved and the non-conserved amino acids, respectively. The orange color indicates the semiconserved residues.

The putative human OCT proteins consist of 12 transmembrane domains (TMDs), intracellular N- and C-termini, one extracellular loop between the first and the second TMD, and one intracellular loop between the sixth and the seventh TMD (Figure 2). Currently, no crystal structure of OCTs has been resolved; hence, the topology and the mode of transport of OCTs are largely based on computational modeling with *E. coli* LacY permease and structure-function characterization. In this model, the binding pocket within the outward-open binding cleft is likely to have overlapping binding sites. The binding of the substrate leads to a series of conformational changes for the release of the substrate into the cytosol. Thereafter, the transporter, empty or loaded with a substrate bound in the inward conformation, can switch back to the outward-open conformation [12,13]. Although most of these studies have been performed on rat Oct1 and Oct2, this mechanistic model is considered to also be valid for the human OCTs. However, the differences between human and rodents concerning substrate selectivity warrant direct structure-function studies on the human OCTs to better understand how these transporters work in humans [14].

OCTs are known as polyspecific transporters because they recognize and transport a broad range of molecules, typically positively charged or zwitterions at physiological pH, such as the organic amines choline and carnitine, the neurotransmitters dopamine and serotonin, the microbiota products trimethylamine (TMA) and trimethylamine N-oxide (TMAO), and the vitamin B1 thiamine [15,16]. OCTs also facilitate the transport of a variety of drugs, including the anticancer platinum derivatives and ifosfamide, the antibiotics gentamicin, cephaloridine and colistin, and the antidiabetic metformin [3,15,17,18]. Ever since their identification, the thermodynamics, kinetics, and substrate specificities of OCTs have been characterized in different overexpressing systems, using prototypical substrates such as radiolabeled tetraethylammonium (TEA) and 1-methyl-4-phenylpyridinium (MPP^+^), and the fluorescent compound 4-[4-(dimthylamino)-styryl]-N-methylpyridinium (ASP^+^). In most cases, OCTs are Na^+^-independent electrogenic transporters, whose activity is driven by the membrane potential across the plasma membrane. Thus, according to the electrochemical gradient of the substrate, OCTs can act as either influx or efflux systems. An exception is represented by OCTN2, which displays a bifunctional mode of transport. OCTN2 transports carnitine and its precursor γ-butyrobetaine in a Na^+^-dependent manner, and other organic cations in a Na^+^-independent manner [19]. OCTs are characterized by different influx kinetics. The OCTN2-mediated L-carnitine uptake seems to follow Michaelis–Menten kinetics [20]. OCT1 and OCT2 appear to have allosteric properties. Koepsell’s group elegantly demonstrated that the rat Oct1 monomer functioned in an allosteric mode [21]. Likewise, our group has shown that the transport of structurally different substrates mediated by the human OCT2 likely involved two cooperative binding sites, suggesting that human OCT2 also had allosteric features [18,22,23].

Expression and localization studies in different species have revealed that OCT1, OCT2, and OCT6 displayed relatively narrow patterns of expression limited to individual organs or tissues. In humans, OCT1 is primarily expressed on the basolateral membrane of enterocytes and hepatocytes (intestines and liver) [24,25], and OCT2 is expressed on the basolateral membrane of proximal tubular cells (kidney) [26]. Initially, OCT6 was considered to be testis specific, as it had been detected only on the luminal membrane of the epididymal epithelium and in the Sertoli cells [27]. Lately, it has also been detected in endometria and in several cancers, suggesting a possible role of OCT6 in cancer resistance [28,29,30,31,32,33,34,35,36,37,38,39]. Noteworthy, in rodents, Oct1 has also been shown to be highly expressed on the basolateral membrane of proximal tubular cells and Oct2 in the brain and inner ear [40,41]. High expression levels in the intestine, liver, and kidneys of OCT1 and OCT2 advocates a cardinal role of these transporters in the intestinal absorption, tissue distribution, and hepatic and renal elimination of several widely prescribed drugs [42]. OCT3, OCTN1, and OCTN2 are more broadly expressed throughout the body [14,20,43,44,45,46]. In polarized epithelia, OCT1, -2, and -3 are restricted to the basolateral membrane. Through a mating-based split-ubiquitin system screening, it has been found that tetraspanin CD63, a four transmembrane domains protein that facilitates cell adhesion and motility, was a protein partner of OCT1, -2 and -3 [47,48]. It has also been demonstrated that CD63 was critical for the correct basolateral localization of OCT2 in proximal tubular cells [47]. The motif sequence that might be involved in the basolateral sorting of OCT1, -2 and -3 is not known. Still, it is noteworthy to highlight the presence of a fully conserved di-leucine sequence, a well-characterized basolateral sorting sequence, in the cytoplasmic tail of these transporters (Figure 2) [49]. Conversely, OCTN1, OCTN2, and OCT6 cellular localization may be tissue dependent. For instance, OCTN2 is expressed on the brush border membrane of enterocytes and proximal tubular cells, and on the sinusoidal membrane of hepatocytes [20,44,45,46,50,51,52,53]. The delivery of proteins to the apical surface most likely depends on multiple coordinated mechanisms, including N-glycosylation pattern, interacting protein partners, and membrane lipid content [49].

As part of the Special Issue Physiology, Biochemistry, and Pharmacology of Transporters for Organic Cations, this article provides a critical overview of the physiological, pharmacological, and toxicological impact and function of organic cation transporters in the key organ systems in which they are expressed. Sources for this review were obtained through extensive literature searches of publications browsing PubMed. Only papers published in the English language were considered.

## 2. Organic Cation Transporters (OCTs) in the Liver

There is a good deal of evidence that OCT1 and OCT3 are expressed in rodent, as well as human liver, whereas OCTN1 and OCTN2 may be expressed in rodent but not in the human hepatocytes. OCT1 represents the most studied hepatic OCT [15]. In this section, we discuss OCT1 primarily and mention some valuable, although not necessarily translatable, animal studies on liver Oct3 and Octn transporters. Oct1 was cloned from the rat in 1994 and found to be highly expressed in the liver and kidney [54]. The human OCT1 was cloned shortly after that and was found in the liver, at the sinusoidal side of hepatocytes, but only marginally expressed in the kidney [24,25]. The *SLC22A1* gene is under the control of the hepatic nuclear transcription factor HNF-4a. When it binds to the promoter region of the *SLC22A1* gene, HNF-4a activates the transcription of the OCT1 mRNA. The HNF-4a-mediated transcriptional activation of the *SLC22A1* gene is inhibited by the bile acid chenodeoxycholic acid, which is the most potent endogenous ligand of the nuclear receptor farnesoid X receptor (FXR) [55]. Indeed, the hepatic expression of OCT1 is lower in patients and animals with cholestasis, a condition in which bile acids accumulate in the liver because of an inefficient elimination in the bile [24,56,57,58]. A number of independent studies have shown that OCT1 expression was also reduced in liver tumors. Although the role of OCT1 in liver carcinogenesis has not been elucidated, it is conceivable that the expression level of OCT1 is likely to determine the pattern of fluorocholine hepatic accumulation, a positron emission tomography (PET) tracer, a substrate of OCT1 that shows promising results in the differential diagnosis of intrahepatic lesions [59,60,61]. At the protein level, OCT1 can be regulated by protein kinase A (PKA) and Ca^++^/calmodulin [62]. Recently, by using rat Oct1 reconstituted in nanodiscs, it has been found that the allosteric binding of rat Oct1 was regulated through interactions with the surrounding lipid microenvironment [63].

Thus far, OCT1 has been primarily characterized from a pharmacological perspective, and its physiological role has only been partially defined. Recently, it has been shown that the total Oct1 knockout mouse, viable with no apparent deficiencies or phenotype, displayed an increased ratio of AMP to ATP, which activated the energy sensor AMP-activated kinase (AMPK), and substantially reduced triglyceride levels in the liver [64]. This phenotype seems to be due to the reduced uptake of thiamine in the Oct1-deficient animals. Thiamine (vitamin B1) is involved in energy transformation pathways as a cofactor of the pyruvate dehydrogenase complex, the α-ketoglutarate dehydrogenase, and the branched-chain α-ketoacid dehydrogenase [65]. Thiamine deficiency compromises the ability of the cell to synthesize ATP, resulting in a constitutive phosphorylation of AMPK, and increased catabolic rate and energy consumption [64,66]. When human OCT1 is expressed in the Oct1^-/-^ mouse, the transgenic liver appears to become prone to steatosis, indicating a role of OCT1 in hepatic lipid and energy metabolism [66]. The pharmacological relevance of OCT1 has been facilitated by the flourishing of pharmacogenomics studies in the last two decades. There is extensive clinical evidence suggesting that the therapeutic effects and toxicity of drugs could be changed in subsets of individuals carrying a certain genetic variant of the *SLC22A1* gene encoding for OCT1. In the *SLC22A1* gene, many nonsynonymous single nucleotide polymorphisms (SNPs) have been identified, some affecting expression or transport activity and others altering substrate selectivity [67]. Genetic variants of the *SLC22A1* gene have been associated with altered pharmacokinetics and pharmacodynamics of several drugs including opioids, the β_2_ agonist fenoterol, and metformin [68,69,70,71,72]. For instance, carriers of the p.Met420del (rs35191146) or p.Arg61Cys (rs12208357) variants, which are associated with decreased transport activity, experienced reduced therapeutic effects, assessed through a glucose tolerance test, as compared with individuals carrying OCT1 wild-type [64,69].

OCT3 colocalizes with OCT1 in the sinusoidal hepatocyte membrane [24]. The role of OCT3 in liver physiology is probably linked to the homeostasis of molecules that are not substrates of OCT1, such as the neurotransmitters adrenaline, noradrenaline, and histamine [14,73]. Studies have shown that the degree of hepatic fibrosis and ductular reaction induced by bile duct ligation or carbon tetrachloride (CCl_4_) treatment was significantly higher in Oct3^-/-^ than wild-type mice, because of an overproduction of TGFβ by stellate cells [74]. As adrenaline and histamine have been shown to promote fibrotic remodeling of the airways and the heart, respectively [75,76], it is possible that the different handling of these neurotransmitters concurs to the excessive hepatic remodeling observed in Oct3^-/-^ mice. Similar to OCT1, the hepatic expression of OCT3 is significantly affected by cholestasis in both humans and rodents; however, the mechanism of transcription repression might be different [24].

OCTN2, encoded by the *SLC22A5* gene, is a high-affinity, Na^+^-dependent, electrogenic carnitine carrier [20]. Carnitine is a vitamin-like compound, highly enriched in red meat or synthesized from γ-butyrobetaine in liver, kidney, and brain [77]. About 25% is synthesized in the body, while the rest is derived from dietary meats [78]. Carnitine is primarily involved in the translocation of mid- and long-chain fatty acids from the cytosol into the mitochondrial matrix, where fatty acid β-oxidation takes place [79]. An important experimental model for the comprehension of carnitine’s physiological role is the juvenile visceral steatosis (jvs) mouse. Jvs mice are characterized by impaired intestinal absorption, tissue distribution, and reabsorption of carnitine, which leads to systemic carnitine deficiency resulting in hepatic steatosis, hypoglycemia, hyperammonaemia, and growth retardation [80]. Shortly after being cloned, Octn2/OCTN2 was found mutated in jvs mice, as well as in patients with systemic carnitine deficiency (OMIM212149) [81]. Notably, OCTN2 can also transport, in a Na^+^-dependent manner, γ-butyrobetaine, the carnitine precursor (K_m_~13 µM) [19]. The role of Ocnt2 in the hepatic uptake of carnitine has been demonstrated in primary cultured mouse hepatocytes, which showed a K_m_ of ~5 µM, consistent with a high-affinity system [52,53]. Carnitine deficiency in the liver, over loss of Octn2, leads to an accumulation of fatty acids in the cytoplasm of hepatocytes. In line with the pivotal role of carnitine in lipid metabolism, the Octn2 expression level is closely linked to lipid homeostasis. The nuclear receptor Pparα, activated by free fatty acids, has been shown to induce the mRNA expression of Octn2 in rodents and pigs in several tissues, including the liver [82,83,84,85,86,87]. Insulin, which positively correlates with fatty acids oxidation in human skeletal muscle [88] has been associated with an increase in carnitine uptake and expression of OCTN2 in skeletal muscle [89]. Taken together, these findings suggest that OCTN2 induction represents an adaptive protective mechanism against lipid metabolism dysfunction.

Mouse Octn1 was found to be expressed in non-parenchymal mouse liver cells, with reports showing functional expression in stellate cells. Upregulation of Octn1 and activation of stellate cells, after treatment with the liver toxin dimethylnitrosamine, were seen to lead to increased liver levels of the natural, nutrient-derived, OCTN1 substrate, antioxidant ergothioneine, which resulted in protection from inflammation, oxidative stress, and more severe liver fibrosis [90]. Although OCNT1 was originally cloned from fetal human liver tissue, neither OCTN1 nor OCTN2 seem to be expressed in adult human liver tissue, although low amounts of mRNA may be detected [91,92]. This highlights the historical difficulties of discerning the physiological and pharmacological relevance of each transporter in humans, in accordance with varying tissue expression patterns.

## 3. OCTs in the Kidney

OCT2 and OCTN2, and to a lesser extent, OCT3 and OCTN1 are expressed in the human kidney. OCT2 and OCT3 are considered to be expressed on the basolateral side of proximal tubule cells [26,93], while OCTN2 and OCTN1 are located (assumed for OCTN1) at the apical brush border membrane [15,94]. The expression pattern of Octs is different in rodents, where Oct1 colocalizes with Oct2 at the basolateral membrane of proximal tubule cells. Mice lacking Oct2^-/-^ are normal, suggesting that the expression of Oct1 alone is sufficient to sustain normal renal function. Because of this functional redundancy, our understanding of the potential role of OCT2 primarily relies on studies that employ mice lacking both Oct1 and Oct2 (Oct1/2^-/-^), which display an impaired tubular secretion of organic cations [95].

OCT2 has been well characterized for its relevance in creatinine tubular secretion, although creatinine has been shown to be a substrate of all the above listed transporters, at least in vitro [96]. Creatinine is largely cleared from the blood by glomerular filtration; however, 10–40% of creatinine is actively secreted into the collecting duct for excretion in proximal tubules [26,97]. OCT2 is deemed to be responsible for the majority of the uptake of creatinine, aided by OAT2 and most likely OCT3, into the tubule cells for subsequent active secretion into the collecting duct over apically (urine-facing)-located SLC47 family members (multidrug and toxin extrusion MATE transporters, MATE1 and MATE2-K) [98,99,100]. The most well-known drugs that lead to transient elevation of serum creatinine through interference at the transporter level with OATs, OCTs, or MATEs are cimetidine, isavuconazole, ranolazine, trimethoprim, vandetanib, probenecid, and pyrimethamine and several antivirals used in the treatment of HIV (dolutegravir, rilpivirine, and cobicistat) [96,101,102,103]. Elevations in serum creatinine under treatment with these compounds do not underlie pathological interruption of kidney function. As serum creatinine is widely used as a diagnostic marker in monitoring nephrotoxicity, drug development relies on the clear delineation between nephrotoxicity and non-pathological transient inhibition of creatinine secretion. Currently, guidance of drug regulatory agencies demands that each molecule in development be tested in vitro for inhibition of OCT2 transport activity in order to predict potential drug–drug interactions [104,105]. An example of a drug–transporter interaction leading to a drug–drug interaction is the reduction in renal metformin secretion by the combined inhibition of MATE and, to a lesser extent, OCT2 and possibly OCT3, by cimetidine [106,107,108,109,110,111,112].

Actual kidney injury mediated by substrates of OCT2 most notably includes anticancer platinum agents, of which cisplatin is the most studied [113]. Cisplatin is a substrate of OCT2, whose toxicity stems from the intracellular accumulation by OCT2-mediated cellular uptake, as seen in rodent models [114,115,116,117,118]. Oct1/2^-/-^ mice are partially resistant to cisplatin-induced nephrotoxicity [40,118,119]. Some protective effects of cimetidine co-application under cisplatin treatment have also been demonstrated in mice [40,117] and humans [120,121], with supporting in vitro evidence [122]. Another very small human study using the OCT2 inhibitor pantoprazole (proton-pump inhibitor) could not ameliorate cisplatin-caused nephrotoxicity in pediatric and adolescent cancer patients [123]. In rodents, it has been indicated that the drug-induced kidney injury incurred by the aminoglycoside gentamicin [18], triptolide [124], and the plant toxin ochratoxin A [125] was dependent on Oct2 expression and function. In vitro data also suggest that the nephrotoxic effects of the antiviral agents defovir, cidofovir, and tenofovir [126], and the anticancer agent ifosfamide also underlie OCT2 uptake [127].

Genetic polymorphisms in OCT2 and OCTN1 have been identified to affect metformin renal excretion, leading to significantly increased peak concentrations and larger serum areas under the curve. On the one hand, patients carrying the OCT2 p.Ala270Ser (rs316019) variant or the OCTN1 p.Thr306Ile (rs272893) may require, similar to those with renal impairment, metformin-dosing reductions [128]. Therefore, it is possible that OCTN1 on the apical membrane is involved in the secretion of metformin into the collecting duct. On the other hand, individuals carrying the OCT2 variant p.Ala270Ser (rs316019), associated with a lower OCT2 activity, benefit from a lower risk of cisplatin-induced nephrotoxicity [115,116].

OCTN2 is physiologically most relevant for the reabsorption of carnitine, where loss or non-functionality of this transporter leads to primary systemic carnitine deficiency through carnitine wasting by renal excretion [78,81,129,130,131]. This has been discussed in the previous section in the context of the liver, because the liver, skeletal muscles, and the heart are tissues that largely rely on fatty acid β-oxidation for energy production, and thus are affected most by carnitine deficiency. However, the underlying cause of primary systemic carnitine deficiency and resulting clinical manifestations also underlies intestinal absorption, as most carnitine is derived from the diet, and, most relevantly, OCTN2-mediated renal reabsorption. Patients with primary systemic carnitine deficiency usually present within the first four years of life with lethargy, irritability, and poor feeding; elevated liver enzymes, hypoketotic hypoglycemia, hyperammonemia, frequently hepatomegaly, and most notably, cardio and skeletal myopathies are observed in these patients. It is further associated with sudden infant death [132]. However, interestingly, some affected persons remain completely asymptomatic into adulthood or present with clinical manifestations of carnitine deficiency only as high fatigability or muscle weakness after exertion or not until metabolically stressed, such as under fasting, diet, or recurrent illness [78,133,134]. Primary systemic carnitine deficiency due to autosomal recessive OCTN2 mutations is treated by oral carnitine supplementation and leads to reduction in clinical manifestations although tissue levels of carnitine seem to remain low [132,135]. Despite the heterogeneous clinical picture for primary systemic carnitine deficiency, it remains clear the OCTN2 in the kidney largely dictates carnitine homeostasis through renal reabsorption, with potentially far reaching clinical implications in energy metabolism throughout the body when dysregulated or lost. Drug-induced systemic carnitine deficiency (and nephrotoxicity) in animals has also been reported. Treatment with colistin (a polymyxin) or cephaloridine (a beta-lactam) is associated with urinary loss of carnitine and systemic carnitine deficiency in rats and rabbits, respectively [136,137,138]. Colistin is transported by human OCTN2 in a Na^+^-independent manner, whereas cephaloridine interaction with OCTN2 is Na^+^-dependent [17,139,140].

## 4. OCTs in the Intestines

OCT1, OCT3, OCTN1, and OCTN2 are expressed in the intestines, where OCT1 and -3 are located at the basolateral membrane of enterocytes and OCTN1 and -2 at the brush border membrane [15,141]. Physiologically, these transporters are likely to contribute, along with other higher affinity uptake transporters, to the intestinal absorption of several dietary substrates. OCT1 and OCT3 may be involved in thiamine uptake at high nutritional concentrations in the intestine [15]. OCT1 and OCT3 might play a role in choline intestinal absorption in rodents but perhaps not in humans, as choline does not seem to be a substrate of the human OCT1 and OCT3 in vitro [59,142,143,144,145]. OCTN2, as the primary carnitine transporter, is also involved in the uptake of dietary carnitine. OCTN1 transports carnitine, although cannot compensate for the loss of OCTN1 in primary carnitine deficiency (addressed in Section 3), and is physiologically more relevant in the uptake of ergothioneine, at least in mice [146,147]. Several OCTN1 and OCTN2 genetic variants, which result in reduced expression or function of the transport protein, have been associated with a susceptibility to inflammatory bowel diseases such as Crohn’s disease, ulcerative colitis, and irritable bowel syndrome [141,148,149,150,151,152,153,154]. Pharmacologically, OCT1 and OCTN1 (and likely OCT3) seem to be involved in the absorption of metformin, with gastrointestinal side effects or intolerance being associated with both OCT1 and OCTN1 reduced or loss-of-function variants [155,156,157,158].

## 5. OCTs in Other Tissues

To briefly summarize the above sections, framed in the context of what is known in terms of pathophysiology and pharmacological relevance, OCT1 plays more significant roles in the liver, while OCT2 and OCTN2 have very important functions in the kidney. The following sections address additional tissues, which are selected based on current research trends. We discuss even less well known or described functions of OCTs in additional (human) tissues, where much of the existing work stems from animal studies. We critically assess current themes in animal studies on OCTs and detail the limited studies on humans for each respective tissue. For a comprehensive summary on the state of knowledge about OCTs in human and rodent models based on tissue expression, we suggest consulting the extensive review by H. Koepsell [15].

### 5.1. Central Nervous System

A rather quickly growing number of studies have addressed Oct2 and Oct3 in regard to their roles in the central nervous system and blood-brain barrier in rodents [159,160,161,162,163,164]. Both transporters transport biogenic amines such as dopamine, epinephrine, norepinephrine, serotonin, and histamine, as well as other neurotransmitters and neuromodulators cyclo(His-Pro), salsolinol, and the l-arginine metabolite agmatine [3]. Oct3 has been found to be massively expressed in circumventricular organs. In addition, while both Oct2 and Oct3 appeared principally expressed in central neurons, Oct3 has also been found in astrocytes, in restricted brain areas such as the dorsomedial hypothalamus nucleus and substantia nigra [161,163], where it has been shown to influence stress-mediated increase in extracellular serotonin levels [162] and neurotoxicity [163]. The roles of Octs in the brain have mainly been examined in Oct2- and Oct3-deficient mice. In vivo, Oct2 invalidation appeared to have preferential consequences on serotonin and norepinephrine uptake and clearance [164], and Oct3 invalidation had more impact on dopamine signaling [161]. Invalidation of Oct2 in mice resulted in abnormal anxiety-related behavior in several conflict paradigms [164]. As compared with wild-type mice, Oct2-deficient mice showed altered sensitivity to the dual serotonin/norepinephrine reuptake inhibitor venlafaxine and the serotonin transporter (SERT) and norepinephrine transporter (NET) inhibitors, citalopram and reboxetine, respectively. Oct2 was recently shown to be an essential modulator of the short- and long-term responses to stress in rodents [165]. Oct2-deficient mice and wild-type mice treated with cimetidine, an OCT2 substrate, were protected from oxaliplatin-induced neurotoxicity [166,167]. This transporter is highly expressed in the limbic and prefrontal cortical regions [164,165], known to control the autonomic and endocrine responses to stress or threats. However, recent expression analyses of OCTs in both mouse and human blood brain barrier samples have revealed negligent to no expression of these transporters at this site [168]. It seems that further studies on the role and expression of OCTs in both rodents and humans are needed to assess the physiological and pharmacological relevance of these transporters in the central nervous system.

### 5.2. Inner Ear

As mentioned in the kidney section of this review, aminoglycoside antibiotics and anticancer platinum agents, most notably gentamicin and cisplatin, respectively, are known for their nephrotoxicity and also to induce irreversible hearing loss. While it cannot be disputed that these agents lead to ototoxicity, since evidence for the relevance of OCT2 in these processes has been presented in both rodents and gineau pigs, literature on humans on this topic is not abundant [169]. Additionally, conflicting evidence has been presented as to the localization of OCT2 in the inner ear structures in the experimental models used [40,170]. A human study with pediatric patients identified the most common OCT2 p.Ala270Ser (rs316019) variant to be protective against ototoxicity under cisplatin treatment. Another very small human study using the OCT2 inhibitor pantoprazole (proton-pump inhibitor) could not ameliorate cisplatin-caused ototoxicity in pediatric and adolescent cancer patients [123].

Interestingly, in humans, mutations in OCTN1 that seemingly affect the correct trafficking of the protein to the apical membrane of stria vascularis endothelial cells, were identified as causative in the screening of consanguineous Tunisian families with autosomal recessive non-syndromic hearing loss. Although the reasons behind the hearing loss were not clear, it was postulated in the study that altered energy status via reduced carnitine uptake in the stria vascularis in such patients may have led to oxidative stress and consequent cell damage resulting in profound hearing loss [171]. It is interesting to note that OCTN1 transports the potent food-derived antioxidant ergothioeine with high affinity, which illicits antioxidant/anti-inflammatory effects [147,172]. It should be noted that the authors of this study emphasized that no other comorbidities (Crohn’s disease or other digestive issues) were reported among the patients assessed. This study provided an example of a quite severe phenotype with the loss-of-function of OCTN1. It would be interesting to assess whether or not this is a population effect in an already challenged patient population and whether or not OCTN2, demonstrating a much higher affinity for carnitine than OCTN1, is expressed in these tissues in humans. The latter is not known and not to be assumed, in the context of this study, should carnitine deficiency in the inner ear, and not the lack of other substrates such as ergothioeine, be the underlying cause for deafness.

### 5.3. Cardiovascular System

Trimethylamine N-oxide (TMAO), which is produced from trimethylamine (TMA) stemming from OCT substrates choline and carnitine from protein and lipid nutrients converted by microbiota in the gut, is associated with cardiovascular disease, and thus considered to be a potential novel pro-atherosclerotic molecule [173,174]. In mice, Oct2 is the major uptake transporter of TMAO, as Oct1/2 knockout mice show highly elevated plasma TMAO levels with reduced renal retention [175,176]. Conversely, the relevance of OCT2 or other OCTs in TMAO handling in humans is still questioned, as TMAO is excreted at a similar rate as creatinine in the human kidney, regardless of age and kidney function, and OCT2 variants are not associated with increased TMAO levels [175,176]. TMAO plasma levels in humans may be indirectly modulated by OCTs, over the uptake in the intestines of dietary nutrients, or directly controlled, in part, over the uptake in the kidney; the contribution of both remain to be elucidated. Interestingly, a choline-TMA lyase small molecule inhibitor has proven to be effective as an anti-atherothrombotic agent by its regulation of host microbe, cholesterol, and bile acid metabolism [177], indicating that inhibition of the conversion of choline, of which OCT2 is the main transporter, to TMA positively impacts cardiac health. In the context of primary systemic carnitine deficiency, oral supplementation of carnitine leads to elevated plasma concentrations of TMAO [178,179,180,181], whereas little information is available on long-term effects on the heart in this patient subset [132]. Several studies have questioned the cardiotoxic effects of TMAO in humans in conjunction with carnitine supplementation [176,177,182,183] and this rather hot topic in cardiovascular health has been extensively reviewed and discussed in recent years [184,185,186,187]. Inducing atherosclerosis in mice usually requires an ApoE^-/-^ or Ldlr^-/-^ genotype, also with or without high-fat diet [188,189]. It might be interesting to assess the endothelial function by organ chamber assay of aortic rings freshly isolated from Oct1/2^-/-^ mice, to further study the role of TMAO, as well as the effects of dietary choline and carnitine handling, on cardiovascular health. It seems clear that more studies in both mice and man are required to fully understand the processes involved in the development of atherosclerosis and the contribution of choline, carnitine, and TMAO transport.

### 5.4. Skeletal Muscle

Response to metformin treatment underlies intestinal (OCTN1 and OCT1), hepatic (OCT1), and renal (OCT2) handling, and also transports into peripheral tissues on the level of the effect on metabolism in both skeletal muscle and adipose tissue. Indeed, the more ubiquitously expressed OCT3 has been implicated, both in mice and man, in the metabolic response to metformin in muscle tissue [107,190]. In addition, in relation to in muscle metabolism, OCTN2 is essential to the distribution of carnitine in muscle tissues and has been shown to be upregulated in muscle tissue in response to insulin [89]. Lack of transport of fatty acids into mitochondria due to insufficient intracellular carnitine levels presents as cardiomyopathies, which are common features of primary systemic carnitine deficiency [81].

### 5.5. Reproductive Organs

OCT6 was cloned in 2002 and directly identified as a carnitine transporter specifically expressed in the human testis in Sertoli cells and epididymal epithelium [5,27], and shortly after in endometria [39]. However, research on this newly identified OCT is ongoing and the physiological relevance in carnitine uptake in reproductive organs over this transporter is unclear. Mentionable from a pharmacological perspective, OCT6 has been found to be differentially expressed in several cancers, several SNPs of which have been associated with pharmacologic implications under treatment with anticancer agents doxorubicin, bleomycin-A5, adriamycin, and cyclophosphamide [28,29,30,31,32,33,34,35,36,37,38].

## 6. Conclusions

The recognition of OCTs as low affinity transporters of frequently prescribed drugs, such as several antibiotics and metformin, the flourishing of pharmacogenetics, and the development of rigorous drug–drug interaction studies for marketing approval have decisively contributed to elucidating the impact of the organ-specific and interorgan functions of these transporters and, in conjunction, their high pharmacological impact. However, with the exception of OCTN2, the understanding of the physio-pathological roles played by OCTs in humans is not fully understood. Comprehension of the roles played by OCTs in physiology is hampered by the partially different substrate specificity and tissue expression between rodents and humans and the lack of obvious phenotypes associated with loss of, or gain of, function of any of these transporters. In general, the phenotype of an organism is not the mere product of its genetic constitution but rather the manifestation of the interaction of the genetic background with various environmental influences. In the study of the physio-pathological role of any gene, the best-case scenario is that the phenotype of a genetic variant is apparent under standard environmental conditions. This is the case of the jvs animals lacking Octn2. Alternatively, the phenotype develops only in specific circumstances that the investigator must understand and optimize. For instance, the potential role of OCTs in the elimination of toxins whose chronic exposure is associated with several aging-related diseases such as Parkinson’s and cardiovascular disease, might suggest that the phenotype of Oct-deficient animals does not manifest just because the animals are not examined in the proper environment, or under the correct challenge or insult, or in the right moment of their life. This seems to be the case for OCT1 in steatosis onset [66] and for OCTN1 and OCTN2 in inflammatory disorders [191,192]. Similarly, the evidence that OCT1 and OCT2 are markedly downregulated in liver and kidney cancer, respectively, may suggest that a chronic impaired function of these transporters might be part of the carcinogenic process [22,59,60,61]. In addition, because frequently prescribed drugs are handled by OCTs, we must be confident that the knowledge gained on these transporters will continue to be highly relevant to drug development and patient care in the future, and will, to some extent, contribute to the understanding of the physiology of the OCTs. To conclude, we summarize this work with the statement that, with the current state of knowledge, it is conceivable, though in part only inferred from tissue expression patterns and functionality in the uptake of endogenous and xenobiotic substrates in vitro, that human OCT1 is relevant in the liver and intestine, OCT2 in the kidney, OCT6 in the reproductive system, and OCTN1, OCTN2, and OCT3 in several tissues, whereby the bulk of knowledge on the latter four transporters is historically less abundant than on the former two.

## Abbreviations

OCT	Organic cation transporter
OCTN	Organic cation transporter novel
SLC	Solute carrier
TMA	Trimethylamine
TMAO	Trimethylamine N-oxide
